# Thermal conductivity of complex materials

**DOI:** 10.1093/nsr/nwz040

**Published:** 2019-03-30

**Authors:** G Jeffrey Snyder, Matthias T Agne, Ramya Gurunathan

**Affiliations:** Department of Materials Science and Engineering, Northwestern University, USA

**Figure 1.**
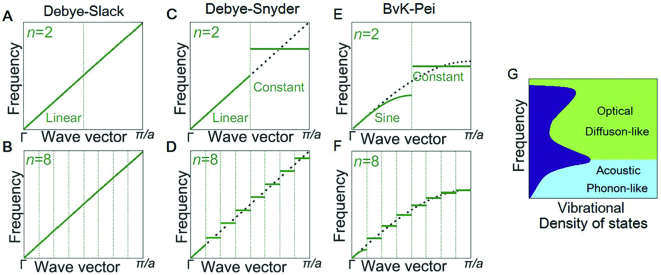
Schematic for a 1D dispersion model used to predict thermal conductivity according to (A) the Debye dispersion used by Slack [2] for a two-atom primitive unit cell and (B) an eight-atom primitive unit cell; compared with (C) the Debye dispersion used by Toberer *et al*. [3] for a two-atom primitive unit cell and (D) an eight-atom primitive unit cell; compared with (E) the BvK dispersion used by Chen *et al*. [1] for a two-atom primitive unit cell and (F) an eight-atom primitive unit cell. In panels C–F, the acoustic branch is treated as phonon-like, whereas the optical branches are treated as more diffuson-like, as illustrated in (G) a schematic vibrational density of states.

In the discussion and analysis of thermal properties (e.g. heat capacity, thermal conductivity) the Debye approximation is typically used to describe the collective behavior of lattice vibrations (phonons) in solid materials. Because the Debye model has been successful in explaining many properties of materials, particularly in contrast to the non-dispersive model considered by Einstein, the Debye model is often the starting point of introductory textbooks in solid state physics. Yet the more we learn about the full spectrum of lattice vibrations, and now can calculate detailed phonon dispersion relations for almost any crystalline solid, the more surprising it is that the linear dispersion Debye model works as well as it has for so long. A real density of vibrational states, for example, appears qualitatively different from the Debye model for all of the many optical branches and even half of the acoustic phonons (Fig. 1G).

In a recent work published in *NSR* by Chen *et al*., ‘Rationalizing phonon dispersion for lattice thermal conductivity of solids’ [1], the effect of a non-linear ‘Born–von Karman’ (BvK) dispersion, which can still be manipulated analytically, is examined on thermal conductivity models. Keeping the speed of the acoustic vibrations (where the Debye model works well) a constant, the result is a subtle decrease in the energy and velocity of the predicted vibrational states (compare Fig. 1A–D with Fig. 1E and F) that leads to a lower predicted thermal conductivity more consistent with experimental values.

The analytic model developed by Chen *et al*. [1] can be compared to a number of simple estimates for the thermal conductivity of complex materials. The ‘Debye–Slack’ model [2] overestimates the thermal conductivity because optical vibrations are treated as acoustic vibrations (Fig. 1A and B). The ‘Debye–Snyder’ (Fig. 1C and D) model [3] treats the optical modes using the Cahill model for the thermal conductivity of a glass and still overestimates the thermal conductivity (note that the minimum optical frequency of ${\omega _{\rm{D}}}{n^{ - 1/d},}$ mentioned by Toberer *et al*. [3], refers to the limits of integration for optical vibrations that range from ${\omega _{\rm{D}}}{n^{ - 1/d}}$ to ${\omega _{\rm{D}},}$ for $d$-dimension considerations).

The ‘BvK–Pei’ model [1] expands on the ‘Debye–Snyder’ model using the BvK rather than the Debye dispersion to give a ∼30% lower estimate due to the lower frequencies and phonon group velocities. Alternatively, one could use a *diffuson* model [4] to account for the optical modes similar to [5], which would also result in a lower estimate for the thermal conductivity.

One possible takeaway from all of this is that maybe Einstein was not as wrong as we make it seem in introductory textbooks. That is, high-frequency optical vibrations in real materials at and above room temperature have a small, but non-negligible, contribution to thermal conductivity. These optical vibrations might actually be better described as more spatially localized (diffuson-like) vibrations [6] than fully delocalized collective (phonon-like) vibrations (Fig. 1G). At high temperatures, despite these profound philosophical differences for the nature and speed of the vibrations, the predicted thermal properties are remarkably similar [4].
